# Comprehensive Evaluation and Analysis of Aging Performance of Polymer-Rich Anchoring Adhesives

**DOI:** 10.3390/ma18153484

**Published:** 2025-07-25

**Authors:** Bing Zeng, Shuo Wu, Shufang Yao

**Affiliations:** CABR Testing Center Company Limited, China Academy of Building Research, Beijing 100013, China

**Keywords:** polymer-rich anchoring adhesives, dynamic thermomechanical analysis, infrared analysis, bending test, alkali, hygrothermal aging

## Abstract

In civil engineering, with the increasing demand for structural reinforcement and renovation projects, polymer-rich anchoring adhesives have attracted much attention due to their performance advantage of having high strength and have become a key factor in ensuring the safety and durability of buildings. In this study, polymer-rich anchoring adhesives underwent three artificial aging treatments (alkali medium, hygrothermal, and water bath) to evaluate their aging performance. Alkali treatment reduced bending strength by up to 70% (sample 5#) within 500 h before stabilizing, while hygrothermal and water-curing treatments caused reductions of 16–51% and 15–77%, respectively, depending on adhesive composition. Dynamic thermomechanical analysis revealed significant loss factor decreases (e.g., epoxy adhesives dropped from >1.0 to stable lower values after 500 h aging), indicating increased rigidity. Infrared spectroscopy confirmed chemical degradation, including ester group breakage in vinyl ester resins (peak shifts at 1700 cm^−1^ and 1100 cm^−1^) and molecular chain scission in unsaturated polyesters. The three test methods consistently demonstrated that 500 h of aging sufficiently captured performance trends, with alkali exposure causing the most severe degradation in sensitive formulations (e.g., samples 5# and 6#). These results can be used to establish quantitative benchmarks for adhesive durability assessment in structural applications.

## 1. Introduction

In civil engineering, structural reinforcement and renovation projects are crucial for ensuring the safety and durability of buildings. Adhesive anchors, as essential connection and reinforcement components, play a pivotal role in these projects [1,2]. In recent years, the utilization of polymer-rich anchoring adhesives in adhesive-type anchor bolts has seen a significant rise. These adhesives exhibit performance advantages such as high strength and excellent durability and are increasingly becoming a focal point for both research and application [3,4].

Polymer-rich anchoring adhesives have the advantages of traditional ones and show properties of rapid consolidation, low fluidity, high-temperature resistance, and low sensitivity to erosive environments [5,6,7]. Polymer-rich anchoring adhesives primarily consist of reactive resins, hardeners, quartz particles, and other inorganic materials [8,9,10]. Polymer-rich anchoring adhesives (such as modified epoxy resin adhesives, modified vinyl ester resin adhesives and non-vinyl ester unsaturated polyester resin adhesive) have exhibited exceptional performance across a wide range of civil engineering applications [11,12]. According to Anam Afzal’s research [13], polymer-rich anchoring adhesives not only possess the same good bonding performance as traditional anchoring adhesives, but their characteristic of a fast consolidation speed could also significantly shorten the construction period, their low fluidity is helpful for precise construction, their high-temperature resistance makes them suitable for structural reinforcement in high-temperature environments, and their low sensitivity to erosive environments enhances the durability of the structure under harsh conditions. In a bridge reinforcement project, A Al-Mosawe [14] pointed out that polymer-rich anchoring adhesives could effectively enhance the bridge structure’s connection strength, improve the structure’s overall bearing capacity, and prolong the service life of the bridge. The structural transformation of high-rise buildings could also provide reliable anchoring connections, thereby ensuring the stability and integrity of the structure [15].

The following methods were adopted to evaluate the performance of anchoring adhesives. The first method involved a bulk specimen test for adhesive material property testing, and a steel–steel bonded single-lap adhesive joint was subjected to a shear test [16,17]. The second method involved conducting a test after implanting screws or rebar into concrete and conducting a test after slicing and artificially aging the rebar-embedded specimens [18,19,20]. The former involved simple sample preparation and testing methods and could be used to quantitatively obtain various physical performance indicators of the adhesive. The conditions of the latter were closer to the actual anchoring application state and could be used to obtain qualitative conclusions, but sample preparation and testing were complex. Both methods have advantages and disadvantages. When the initial single-shear test method was applied to evaluate the durability of polymer-rich anchoring adhesives, interfacial failure (adhesive–steel bond breakdown) often preceded cohesive failure (internal adhesive fracture) due to the polymer’s properties. This premature interfacial failure skewed the test outcomes, preventing an accurate assessment of the adhesive’s true load-bearing capacity, as its structural integrity was not fully challenged. When the second method, whose conditions were closer to the actual working conditions, was used for the test, even if the short-term accelerated aging method (for example, thermal aging, wet heat aging, and fluorescent ultraviolet aging) was adopted for testing, it was still very difficult to truly simulate the long-term aging effect in the actual environment, and only a phenomenological assessment could be carried out.

The selection of aging test conditions aimed to replicate the actual complex environment and ensure reliable and valid results [21]. The design of an alkaline medium environment simulated the internal conditions of concrete. In real-life engineering applications, the alkali compounds generated from the hydration products of concrete elevate the pH value [22,23,24]. Highlighted in the research by Allan Manalo et al. [18], the high alkalinity of the pore solution within concrete created an erosive environment for reinforcing bars, potentially compromising the integrity of the adhesive interface. The damp and hot environment replicated the conditions that buildings experienced under natural high-temperature and high-humidity settings, such as those found in tropical rainforests or coastal areas [25,26]. Francesco Ascione et al. [27] demonstrated that a hot and humid environment accelerated the aging process of epoxy adhesives. A long-term experiment was conducted under conditions of 98% humidity and a seawater environment for 15 months, revealing a trend in performance changes and providing a foundation for evaluating durability. A water bath environment was used for special engineering scenarios such as water conservancy projects [28,29]. Lai et al. [30] conducted a durability study in which externally bonded CFBR concrete beam specimens were exposed to three elevated water temperatures (25 °C, 40 °C, and 60 °C). The results showed that in the water baths at 40 °C and 60 °C, the bonding layers of all specimens gradually deteriorated.

This is a comprehensive comparative study on polymer-rich anchoring adhesives under three aging treatments: alkali treatment, heat–moisture treatment, and water-curing treatment. This study simultaneously used a bending test, dynamic thermomechanical analysis (DMA) tests, and infrared spectroscopy (IR) analysis tests to explore the aging performance of the adhesives in depth. Previous studies were mostly limited to a single aging treatment or a few testing methods. This study provided a new perspective and method for comprehensively evaluating the aging performance of such adhesives. Through the comprehensive application of these testing methods, this study systematically evaluated the effectiveness of various artificial accelerated aging protocols—including test durations and methodologies—for assessing polymer-rich anchoring adhesives. The findings provided robust theoretical and technical foundations for their rational application and durability assessment in engineering practice. Furthermore, this work addressed current research gaps in aging performance evaluation, thereby advancing the understanding of failure mechanisms in polymer-modified adhesive systems. This study helped to ensure the long-term safety and stability of various engineering structures, reducing the risk of structural failures caused by adhesive aging and improving the overall quality and service life of engineering projects.

## 2. Experimental Section

### 2.1. Experimental Materials

Six kinds of polymer-rich anchoring adhesives filled with fillers were selected, including three kinds of modified epoxy resin adhesives (the sample numbers were 1#, 3#, and 5#), two kinds of modified vinyl ester resin adhesives (the sample numbers were 2# and 4#), and one kind of non-vinyl ester unsaturated polyester resin adhesive (the sample number was 6#). These six types of anchoring adhesives were extensively utilized across various sectors of construction engineering.

The main chemical components of modified epoxy resin adhesives are bisphenol A epoxy resin, bisphenol F epoxy resin, polyamide/amine curing agents, and liquid polysulfide rubber/acrylate. The main chemical components of modified vinyl ester resin adhesives are bisphenol A vinyl ester resin, acrylate, and benzoyl peroxide. The main chemical components of non-vinyl ester unsaturated polyester resin adhesive are phthalic acid-type unsaturated polyester, styrene, and benzoyl peroxide.

### 2.2. Experimental Instruments

The test instruments used in this study and the manufacturers they were sourced from are shown in Table 1 below.

### 2.3. Experimental Preparation

**Figure 1:**
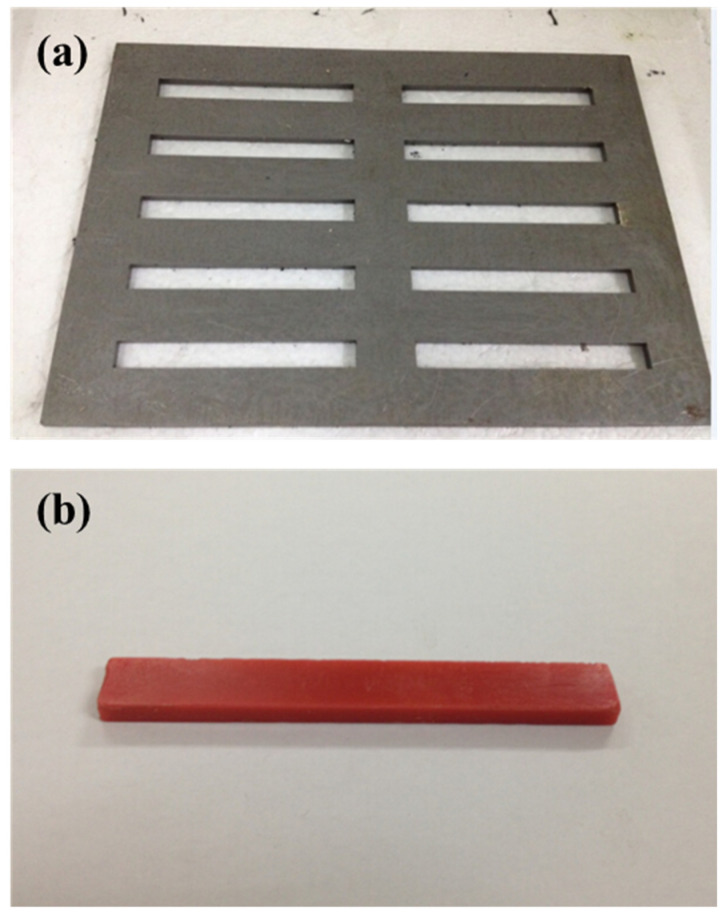
Steel mold (**a**) and sample (**b**).

The adhesive bulk specimens were prepared according to the requirements of GB/T-2567-2021 (*Test methods for properties of resin casting body*) [31]. They were cast and molded using a steel mold with an inner cavity size of 80 × 10 × 4 mm, as shown in Figure 1. After thoroughly mixing the adhesive in accordance with the mixing ratio provided by the supplier, it was processed with a non-intrusive material homogenizer to minimize the formation of air bubbles. Subsequently, it was filled into the steel mold coated with a release agent using an adhesive gun with a syringe. After being placed under standard conditions (23 °C, 50% humidity) for 24 h, the specimens were de-molded. The molds were removed, and the specimens were further cured under standard conditions for 6 days. Every four specimens were grouped, with three of them used for physical property tests and one for structural property tests. Each adhesive was prepared with thirteen groups of samples, with a group used for the standard state test and four groups each for the other three artificial aging tests.

Among the factors affecting the durability of the adhesive, three representative artificial aging simulation test methods, namely those using an alkaline medium, heat–moisture environment, and water-curing environment, were selected for the durability test. The specific test conditions and sampling periods were as follows.

For the alkali treatment test, the oven temperature was set at 35 °C with forced air circulation. The specimens were placed in an alkali medium container, sealed, and then placed in the oven. The alkali medium was a saturated solution of Ca(OH)_2_ with a pH value of 13.2 ± 0.2. The sampling periods were 500 h, 1000 h, 1500 h, and 3000 h.

For the heat–moisture treatment test, the temperature was 50 °C, and the humidity was 98%. The sampling periods were 500 h, 1000 h, 1500 h, and 3000 h.

For the water-curing treatment test, the temperature was 80 °C. The sampling periods were 72 h, 168 h 360 h, and 480 h.

### 2.4. Testing and Characterization

(1) Bending property test of adhesive

There are many physical property indicators and test methods used for adhesives, such as splitting tensile, bending, compressive, tensile–shear, etc. In this experiment, the bending strength test method, which was the most sensitive to polymer adhesives, was used to evaluate the attenuation of the bending strength of the adhesive under the influence of different aging durations.

**Figure 2:**
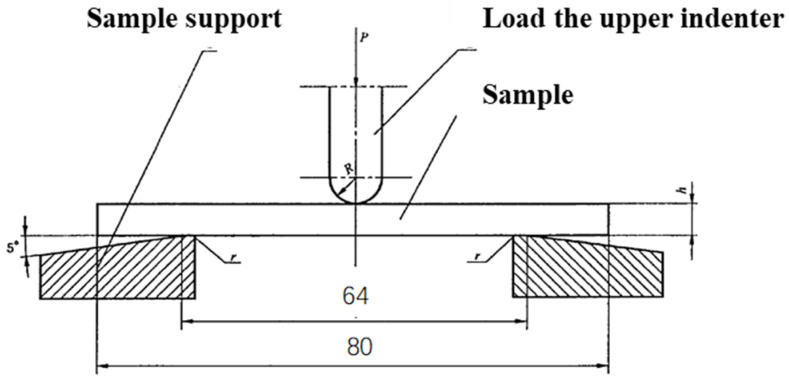
A schematic illustration of the bending strength test for the adhesive.

The bending strength test of the adhesive specimen was carried out by placing the specimen on a three-point test device (see Figure 2), with a span of 64 mm. The specimen was loaded at a speed of 5 mm/min until it failed, and the failure load or the maximum load value was recorded.

To ensure the accuracy and repeatability of the bending test, five samples are selected for testing each time. The average value of the bending strength is taken, and it is ensured that the error value is within 5%.

(2) Dynamic thermomechanical analysis test

**Figure 3:**
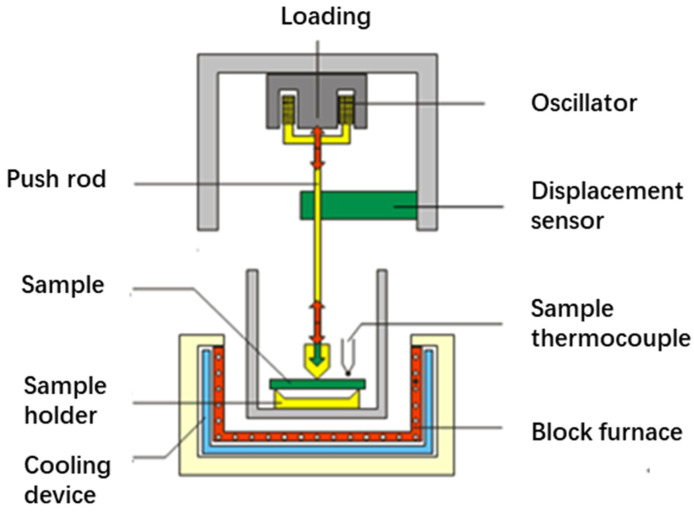
A schematic diagram of the basic principle of the DMA.

The basic principle of dynamic thermomechanical analysis is illustrated in Figure 3. During the programmed temperature process (linear heating, cooling, constant temperature, their combinations, etc.), a single-frequency or multi-frequency periodic sinusoidal fluctuating dynamic oscillation force was applied to the sample, causing the sample to produce a corresponding periodic fluctuating deformation [32,33]. Through mathematical calculation, the DMA spectrum of the loss factor curve could be obtained. Based on the relationship between the loss factor and temperature after artificial accelerated aging treatment, the aging attenuation degree of the adhesive was evaluated. The magnitude of the loss factor represented the viscoelastic properties of the material [34]. The loss factor is inversely correlated with the elastic dominance of a material, whereas a higher value indicates stronger viscous behavior. Dynamic mechanical analysis was conducted in three-point bending mode under a frequency of 1 Hz, with temperature ramping from ambient to 200 °C at a heating rate of 5 K/min. The specimen size was 40 mm × 10 mm × 4 mm. Three samples were used, and the average value was taken for the calculation of the loss factor, with an error within 5%.

(3) Infrared spectroscopy analysis test

The application of infrared spectroscopy could be used to analyze the changes in the functional groups of the molecular weight structure of the standard adhesive [35,36,37]. Fourier transform infrared spectroscopy was employed to characterize functional group variations in the adhesives before and after aging. A comprehensive quantitative–qualitative evaluation was conducted to assess the impact of distinct artificial accelerated aging protocols on the degradation behavior of polymer-rich anchoring adhesives with varying formulations. The measured wavelength range was 400–4000 cm^−1^.

## 3. Results and Discussion

### 3.1. Test Results of Physical Performance

**Figure 4:**
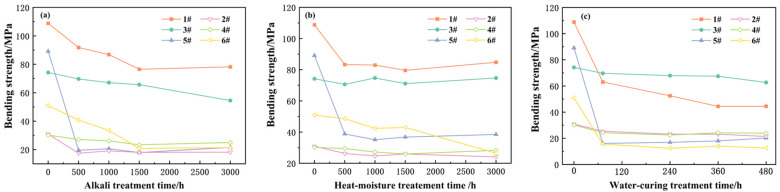
Bending strength variation in different polymer-rich anchoring adhesives under three aging treatments ((**a**) alkali treatment, (**b**) heat–moisture treatment, (**c**) water-curing treatment).

Figure 4 illustrates the temporal evolution of the normalized bending strength for various types of polymer-rich anchoring adhesives following three distinct artificial aging treatments.

With regard to the alkali treatment, as shown in Figure 4a, the bending strength of the polymer-rich anchoring adhesives exhibited a decreasing trend as the treatment duration increased. Notably, within the initial 500 h of the alkali treatment, the bending strength of most adhesives decreased significantly. However, after the treatment time exceeded 1500 h, the decreasing trend in the bending strength became noticeably slower and tended to stabilize.

In the context of the heat–moisture treatment, as shown in Figure 4b, different adhesives behaved differently. Some adhesives (1# and 3#) were able to maintain a relatively stable bending strength within the initial 500 h. However, the bending strength of some other adhesives decreased sharply during this period. After 500 h, the bending strength of most anchoring adhesives no longer continued to decline. However, the unsaturated polyester resin-based anchoring adhesive exhibited a unique variation pattern, with its bending strength showing a significant decrease after more than 1500 h of heat–moisture aging treatment.

The changing trend in the adhesive’s bending strength under the water-curing treatment was similar to that under the heat–moisture treatment. As shown in Figure 4c, the adhesives that exhibited sensitivity during the heat–moisture treatment also showed sensitive characteristics under the water-curing treatment. The change law of their bending strength could be basically clearly determined after 3 days of water-curing treatment.

Through a comparative analysis of the results of the three aging treatments, it could be seen that the alkali treatment had a significant impact on the bending strength of the adhesive in the early stage, and the impact tended to weaken in the later stage. The effects of heat–moisture aging and water-curing aging within 500 h varied depending on the type of adhesive. Among them, the performance change in the unsaturated polyester resin-based adhesive after more than 1500 h of heat–moisture aging was worthy of in-depth study. This might be related to its unique chemical structure and composition, providing important clues for further exploring the performance evolution mechanism of polymer-rich anchoring adhesives in different aging environments.

Based on the degree of decrease in bending strength, it could be basically judged that the polymer epoxy resin adhesive sample 3# and the modified vinyl ester resin adhesive sample 4# had relatively low sensitivities to several aging treatments. Meanwhile, the other four samples all showed different degrees of decrease under different aging treatments.

**Figure 5:**
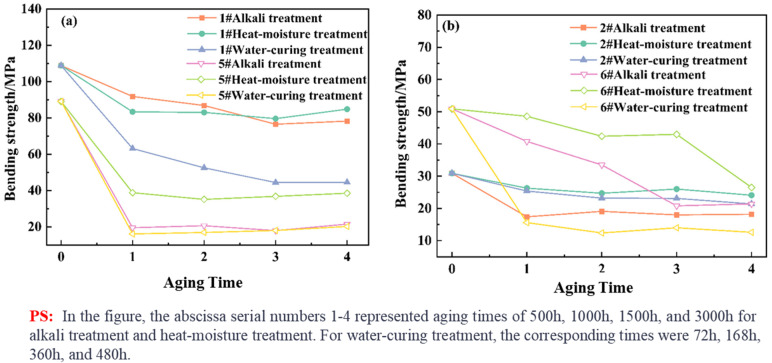
(**a**,**b**) The relationship of the bending strength of various adhesives with time under different aging treatments.

Figure 5 presents the relationship curves of the adhesives with more obvious decreases in bending strength with aging time under different aging treatment environments. It could be seen that for the epoxy resin adhesive sensitive to aging treatment, its aging degree could be judged according to the degree of strength decrease after 500 h of aging treatment. However, after 500 h, regardless of the aging treatment method and the treatment time, the decrease in strength was no longer obvious, and it was difficult to make a judgment on the aging degree again. That is to say, the aging tended to stabilize. There was a similar rule for the vinyl ester-based anchoring adhesives. The strength decreased significantly after 500 h of aging treatment, but the decrease in strength was not obvious after 500 h. At the same time, it was also noted that although the bending strength of the second group of vinyl ester-based anchoring adhesives was not highly sensitive to heat–moisture treatment and water-curing treatment, its bending strength still decreased significantly under the alkali treatment environment. For the unsaturated resin-based anchoring agents, the bending strength decreased with an increase in alkali treatment aging time and tended to stabilize after 1500 h. Meanwhile, for the other two aging treatments, it also tended to stabilize after 500 h.

It could be seen from this that when using the bending strength to determine the aging performance of polymer-rich anchoring adhesives, the methods of 500 h alkali treatment, heat–moisture treatment, and water-curing treatment could be used for inspection. Although the bending strength of some anchoring adhesives was not sensitive to heat–moisture treatment or water-curing treatment, it would have a relatively large decrease under alkali treatment artificial aging.

Subsequently, the performance aging rates of the six materials in three artificial accelerated aging environments were determined using Formula (1). The performance aging rates of the adhesives under the three different aging treatment methods are summarized in Table 2.

(1) $$P=\frac{\left|\sigma_{1}-\sigma_{0}\right|}{\sigma_{0}}\times 100\%\;$$

Among these parameters, *P* represents the performance aging rate, σ_1_ denotes the bending strength of the sample post-aging, and σ_0_ indicates the bending strength of the sample pre-aging.

Through the research on the aging of the physical properties of polymer-rich anchoring adhesives, the following conclusions were drawn: Under the three aging methods of alkali treatment, heat–moisture treatment, and water-curing treatment, the aging rates of the mechanical properties (bending strength) of the samples showed different rankings. For samples 1#, 2#, 5#, and 6#, the aging rate after water-curing treatment was the highest, followed by alkali treatment, and the lowest was obtained after heat–moisture treatment. For samples 2# and 3#, the aging rate of alkali treatment was the highest, followed by water-curing treatment, and the lowest was obtained for heat–moisture treatment. This difference was closely related to the curing system of the adhesive products. Different curing systems caused the products to have different aging degrees in different aging environments. Therefore, when evaluating the aging performance of products, it was not possible to rely solely on the results of a single aging test. Instead, the mechanical properties after multiple aging treatments should be comprehensively considered to ensure the accuracy and comprehensiveness of the evaluation.

### 3.2. Test Results of Dynamic Thermomechanical Analysis

After analyzing the physical performance of the adhesives, the dynamic thermomechanical behavior of the materials was further investigated through DMA tests.

**Figure 6:**
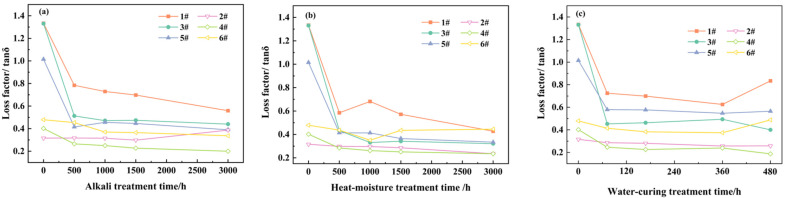
Comparative chart of loss factor of various polymer-rich anchoring adhesives over time under different aging treatments ((**a**) alkali treatment, (**b**) heat–moisture treatment, (**c**) water-curing treatment).

Figure 6 illustrates the variation in the maximum loss factor of the polymer-rich anchoring adhesives under three distinct aging treatment methods as a function of treatment time.

It was observed that under normal temperature conditions, epoxy adhesives typically exhibited a relatively high loss factor, with standard state values exceeding 1.0. After undergoing three different aging treatments for 500 h, the maximum values of the loss factor showed a significant decrease and subsequently stabilized without substantial changes. This phenomenon suggested that in the standard state, the degree of cross-linking within the molecular chains of epoxy adhesives was insufficient, leading to a higher proportion of flexible groups [38]. As the aging process progressed, the material’s rigidity gradually increased until it reached a stable state.

In contrast, the maximum loss factors of the modified vinyl ester resin adhesives and the non-vinyl ester unsaturated polyester resin adhesives at room temperature were generally at a relatively low level, ranging from 0.3 to 0.5 in the standard state. This meant that in the standard state, the rigid components of the groups on their molecular chains dominated [39]. After 500 h of aging treatment, aside from the fact that the maximum loss factor of 4# sample did not change significantly under the alkali treatment environment, the adhesives in the other treatment environments all showed a significant decrease in the maximum loss factor, and the decreasing trend gradually slowed down with the extension of time. This change trend implied that the rigidity of the adhesive increased, and the brittleness also increased, leading to a decrease in mechanical properties. It was worth noting that the maximum loss factor of the unsaturated 4# sample hardly changed after alkali treatment, and its decreasing trend in the heat–moisture aging and water-curing treatment processes was significantly more gradual than that of other unsaturated adhesives or epoxy adhesives. This fully demonstrated that due to the differences in the main agents and curing systems, different aging methods significantly influenced the structure and performance of the cured adhesives.

In summary, the DMA test clearly elucidated the variation patterns of loss factors for different types of adhesives during the aging process. This provides a critical foundation for understanding their aging mechanisms and assessing the impact of various aging methods, thereby facilitating a deeper comprehension of the aging performance of polymer-rich anchoring adhesives.

### 3.3. Test Results of Infrared Spectroscopy Analysis

In the evaluation of the aging performance of polymer-rich anchoring adhesives, the IR analysis revealed the changes in the molecular structure of the adhesives under different aging treatment methods at the microscopic level. The following discussion was carried out for three different types of anchoring adhesives (1#, 4#, and 6# samples) according to the three aging treatment methods of alkali treatment, heat–moisture treatment, and water-curing treatment.

#### 3.3.1. Results of Alkali Treatment

**Figure 7:**
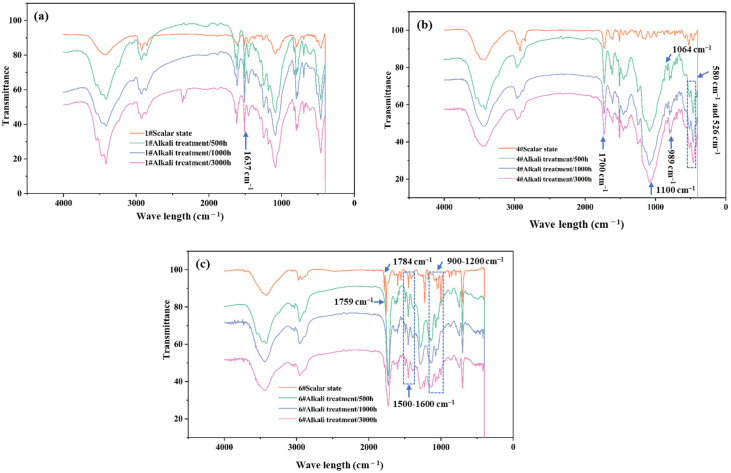
Comparison of infrared spectra of 1# epoxy resin adhesive (**a**), 4# modified vinyl ester resin adhesive (**b**), and 6# non-vinyl ester unsaturated polyester resin adhesive (**c**) before and after alkali treatment.

For the 1# epoxy resin adhesive (Figure 7a), after alkali treatment, a new peak appeared at 1637 cm^−1^. This phenomenon indicated that under the high-temperature condition of alkali treatment, C-O underwent an oxidation reaction to form C=O, and at the same time, the epoxy group underwent a hydrolysis reaction under the action of OH^−^, resulting in the degradation of the adhesive molecular chain. However, from the overall infrared spectrum, the degree of change was not very significant.

In the infrared spectrum of the 4# modified vinyl ester resin adhesive (Figure 7b), the intensities of the C=O stretching vibration peak at 1700 cm^−1^ and the C-O stretching vibration peak at 1100 cm^−1^ changed significantly before and after alkali treatment, which meant that the ester functional group was broken during the alkali treatment process and was the first part to be affected. In addition, the peak at 989 cm^−1^ disappeared, the peak at 580 cm^−1^ was significantly weakened, the peak at 1064 cm^−1^ shifted to a higher frequency, and the peak at 526 cm^−1^ shifted to a lower frequency.

For the 6# non-vinyl ester unsaturated polyester resin adhesive (Figure 7c), the infrared spectrum after alkali treatment showed significant changes. The peak at 1784 cm^−1^ disappeared, and the peak at 1759 cm^−1^ shifted to a lower wavenumber, indicating that the ester group was damaged. The peak shapes in the ranges of 1500–1600 cm^−1^ and 900–1200 cm^−1^ changed significantly, and double peaks at 1286 cm^−1^, 1158 cm^−1^, and 1131 cm^−1^ were generated. At the same time, the peaks at 1552 cm^−1^, 1227 cm^−1^, 1177 cm^−1^, and 998 cm^−1^ disappeared, showing that its structure had undergone a large change.

The influence of alkali treatment on the molecular structures of different adhesives was significantly different. Although the molecular structure of the 1# epoxy resin adhesive changed to a certain extent, the overall change range was relatively small. In the 4# modified vinyl ester resin adhesive, the ester functional group was greatly affected by alkali treatment. This structural change was consistent with the trend of the mechanical properties of this adhesive continuously decreasing slightly during alkali treatment, indicating that structural changes, such as the breakage of the ester functional group, had a negative impact on its mechanical properties. The molecular chain of the 6# non-vinyl ester unsaturated polyester resin adhesive might be broken under alkali treatment, and the structure was severely damaged. This observation described a scenario in which the mechanical properties exhibited a substantial linear degradation pattern (showing a 60% reduction in performance metrics) during the initial 1500 h exposure period. The established correlation offered a plausible mechanistic explanation for the property alterations through microstructural analysis while also demonstrating the critical interdependency between alkali-induced modifications in the adhesive’s molecular architecture and the corresponding mechanical performance deterioration.

#### 3.3.2. Results of Heat–Moisture Treatment

**Figure 8:**
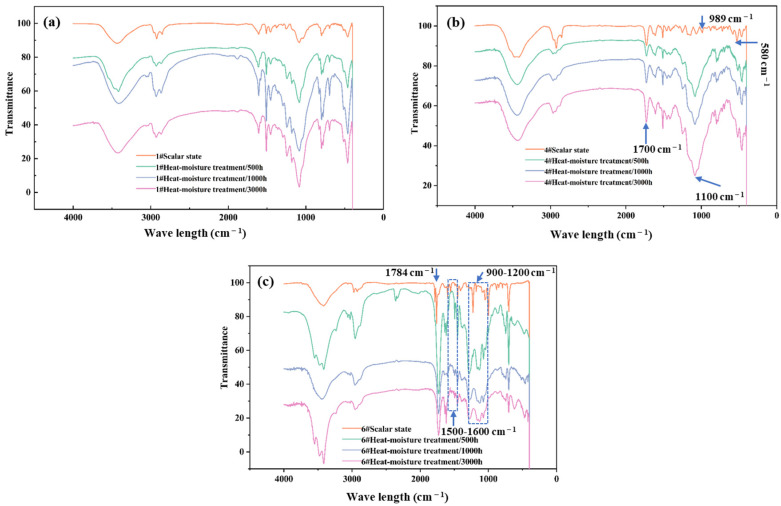
Comparison of infrared spectra of 1# epoxy resin adhesive (**a**), 4# modified vinyl ester resin adhesive (**b**), and 6# non-vinyl ester unsaturated polyester resin adhesive (**c**) before and after heat–moisture treatment.

As shown in Figure 8a, after heat–moisture treatment, the infrared spectrum of the 1# epoxy resin adhesive remained basically unchanged, and no obvious increase or decrease in peaks or peak shift phenomenon occurred. However, as shown in Figure 8b, after heat–moisture treatment, the intensities of the C=O stretching vibration peak at 1700 cm^−1^ and the C-O stretching vibration peak at 1100 cm^−1^ of the 4# modified vinyl ester resin adhesive were significantly enhanced, which indicated that the ester functional group was broken during the heat–moisture treatment process. At the same time, the peak at 989 cm^−1^ disappeared, and the peak at 580 cm^−1^ was significantly weakened, but the overall molecular structure did not change significantly. For the 6# non-vinyl ester unsaturated polyester resin adhesive (Figure 8c), the change in the infrared spectrum after heat–moisture treatment was similar to that after alkali treatment. The peak at 1784 cm^−1^ disappeared, and the peak at 1759 cm^−1^ shifted to a lower wavenumber, indicating that the ester group was partially damaged. The peak shapes in the ranges of 1500–1600 cm^−1^ and 900–1200 cm^−1^ changed significantly, and the structural change was relatively obvious.

The influence of heat–moisture treatment on the molecular structure of the 1# epoxy resin adhesive was minimal, which was highly consistent with the change law of its mechanical properties, with a slight decrease at 500 h and almost no change thereafter after heat–moisture treatment, indicating that its structural stability was good and it could resist the influence of the heat–moisture environment. Under heat–moisture treatment, the 4# modified vinyl ester resin adhesive mainly had changes such as the breakage of the ester functional group, and the influence on the overall molecular structure was limited. This was consistent with the situation in which the mechanical properties of this adhesive hardly changed after heat–moisture aging, once again proving the consistency between the infrared spectrum analysis results and the mechanical property test results. The molecular structure of the 6# non-vinyl ester unsaturated polyester resin adhesive was significantly damaged under heat–moisture treatment, which explained the phenomenon of its mechanical properties decreasing linearly and significantly (with a decreased range of 40%) after heat–moisture aging well, indicating that the influence mode and degree of heat–moisture treatment on the molecular structures of different adhesives were different. The change in the infrared spectrum could accurately reflect the internal mechanism of the performance change in the adhesive during heat–moisture aging and provided a powerful basis for the in-depth understanding of the influence of heat–moisture aging on the adhesive.

#### 3.3.3. Results of Water-Curing Treatment

**Figure 9:**
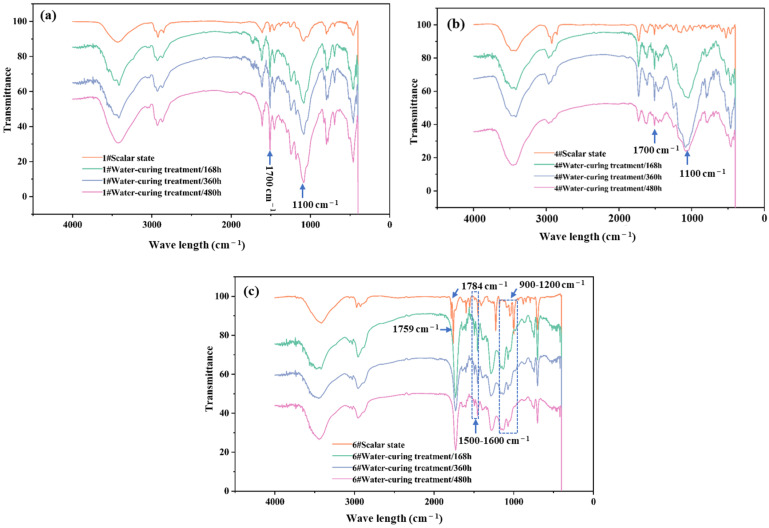
Comparison of infrared spectra of 1# epoxy resin adhesive (**a**), 4# modified vinyl ester resin adhesive (**b**), and 6# non-vinyl ester unsaturated polyester resin adhesive (**c**) before and after water-curing treatment.

After water-curing treatment, for the 1# epoxy resin adhesive (see Figure 9a), the intensities of the C=O stretching vibration peak at 1700 cm^−1^ and the C-O stretching vibration peak at 1100 cm^−1^ were both significantly enhanced, but there was no increase in or disappearance of peaks, indicating that its molecular structure did not change significantly. For the 4# modified vinyl ester resin adhesive before and after water-curing treatment (see Figure 9b), the intensities of the C=O stretching vibration peak at 1700 cm^−1^ and the C-O stretching vibration valley point at 1100 cm^−1^ were enhanced, which meant that the ester functional group was broken during the water-curing treatment process. At the same time, the peak at 989 cm^−1^ disappeared, and the peak at 580 cm^−1^ was significantly weakened, but the overall change in the molecular structure was relatively small. The infrared spectrum of the 6# non-vinyl ester unsaturated polyester resin adhesive after water-curing treatment changed significantly (see Figure 9c). Compared with the standard state, the peak at 1784 cm^−1^ disappeared, and the peak at 1759 cm^−1^ shifted to a lower wavenumber, indicating that the ester group was partially damaged. The peak shapes in the ranges of 1500–1600 cm^−1^ and 900–1200 cm^−1^ changed significantly, and the structural change was relatively large.

The influence of water-curing treatment on the molecular structure of the 1# epoxy resin adhesive was relatively small, which was consistent with the relatively stable mechanical performance of this sample after water-curing aging, indicating that sample 1# could maintain the integrity of its molecular structure well in the water-curing environment and thus maintain the stability of mechanical properties. During the water-curing treatment process of the 4# modified vinyl ester resin adhesive, although the ester functional group had changes such as breakage, the influence on the overall structure and mechanical properties was not significant, further proving the consistency between the infrared spectrum analysis results and the mechanical property test results. The molecular chain of the 6# non-vinyl ester unsaturated polyester resin adhesive might be broken under water-curing treatment, and the structural change was obvious. This corresponded to the situation where the mechanical properties decreased significantly (with a decreased range of 60%) at 500 h and hardly changed thereafter, reflecting the specificity of the influence of water-curing treatment on the molecular structures of different adhesives. The change in the infrared spectrum could reasonably explain the influence of water-curing aging on the adhesive performance from a microscopic perspective and was helpful for an in-depth exploration of the internal reasons for the performance change in the adhesive during water-curing aging.

### 3.4. Comparison of Results of Three Test Methods

In the evaluation of the aging performance of polymer-rich anchoring adhesives, three methods, namely the physical property test, DMA test, and IR analysis test, were used. The following is a comprehensive comparative analysis of the results.

The physical property test used bending strength to measure the change in mechanical properties during aging. For some aging-sensitive adhesives (such as some epoxy and vinyl ester types), after 500 h of aging treatment, the change in bending strength could be used to judge the aging degree, and strength tended to stabilize. The DMA test showed that at room temperature, the standard state loss factor of epoxy adhesives was high. After 500 h of aging treatment, it decreased significantly and then tended to stabilize, which was consistent with the change law of bending strength. This indicated that aging caused a cross-linking change in the molecular chains of epoxy adhesives and an enhancement in rigidity. The standard state loss factor of unsaturated resin adhesives was low. After 500 h of aging treatment, it decreased except in special cases, and then the decreasing trend weakened, which was in line with the change law of the bending strength (except for the special case of alkali treatment), reflecting the internal relationship between the results of the two test methods.

Different aging treatments had different effects on the bending strength of the adhesive. In the early stage of alkali treatment, it significantly affected the bending strength of some adhesives (such as unsaturated resin types) and then tended to stabilize after 1500 h. The heat–moisture and water-curing aging treatments had an obvious effect on some adhesives within 500 h and then tended to stabilize (the unsaturated polyester resin type had a special change after more than 1500 h of heat–moisture aging). The IR analysis selected the 1# epoxy resin adhesive, the 4# modified vinyl ester resin adhesive, and the 6# non-vinyl ester unsaturated polyester resin adhesive for research. After alkali treatment, the molecular chain of the 1# epoxy resin adhesive was degraded, which was consistent with the decrease in bending strength. After heat–moisture treatment, the infrared spectrum was consistent with the change in mechanical properties. After the water-curing treatment, the molecular structure was in line with the change in mechanical properties. After the alkali treatment of the 4# modified vinyl ester resin adhesive, the breakage of the ester functional group was related to the decrease in mechanical properties. After heat–moisture treatment, the change in the ester group was consistent with the change in mechanical properties. After the water-curing treatment, the change in the functional group was consistent with the overall structure and mechanical property change. For the 6# non-vinyl ester unsaturated polyester resin adhesive under the three aging treatments, the changes in the functional groups and structure in the infrared spectrum corresponded to the changes in mechanical properties.

The DMA test reflected the change in the viscoelastic properties of the material through the change in the loss factor, providing macroscopic information for evaluating the degree of aging attenuation. The IR analysis focused on the microscopic changes in the functional groups and explained the essential reasons for aging. The two test methods complemented each other. The macroscopic results provided a basis for the microscopic explanation, and the information on the microscopic structural changes helped us to understand the mechanism of the change in the macroscopic loss factor.

By integrating the results from IR spectroscopy, DMA, and bending tests, a robust micro-to-macro performance correlation model can be established: chemical bond degradation (as detected by IR spectroscopy) induces alterations in molecular chain mobility (observed via DMA), ultimately leading to variations in macroscopic mechanical properties (evaluated through bending tests). Specifically,

1. Chemical bond changes dominate performance evolution: The extent of ester group (C=O and C-O) cleavage directly determines the adhesive’s anti-aging ability. Sample 6# has the most severe ester group damage and the most significant performance decline.
2. Molecular chain movement ability reflects aging state: The change in loss factor reveals the variation in molecular chain segment movement ability. An initially high loss factor (such as in epoxy resin) indicates the free movement of molecular chain segments. A decrease in the loss factor after aging indicates an increase in molecular chain rigidity, which is directly related to the decline in flexural strength.
3. Microscopic explanation of the performance stabilization period: When the IR spectrum indicates that the main functional groups no longer change significantly and the DMA loss factor stabilizes, the adhesive network structure reaches a new equilibrium state, which is macroscopically manifested by the flexural strength no longer declining.

Based on the integrated results from IR spectroscopy (detecting chemical bond degradation), DMA (quantifying molecular chain mobility changes), and bending tests (measuring mechanical strength decay), this study establishes a definitive micro–macro correlation for adhesive aging behavior. These material-specific insights enable targeted engineering decisions: the 4# sample serves as the optimal choice for concrete bridges due to its alkali resistance (19% aging rate), while 3# excels in hydraulic structures with 15% water-aging resistance. For temporary applications, cost-efficient 6# may be used but avoided in humid conditions due to 51% humidity-induced degradation. This multi-method framework translates fundamental aging mechanisms into verified selection criteria for structural safety and durability optimization. This science-based selection approach transforms adhesive procurement from a commodity decision into a capital allocation strategy, demonstrating how molecular-level insights drive macroeconomic outcomes.

## 4. Conclusions

1. Aging Rate and Performance Evaluation

Polymer-rich adhesives exhibited distinct degradation patterns under different aging treatments, with water-curing causing the most severe deterioration (15–77% strength loss). Alkali treatment drove significant early-stage degradation (e.g., 70% loss in epoxy 5# at 500 h), while hygrothermal aging showed material-specific effects (vinyl ester 4# maintained <1% loss). Unsaturated polyester 6# proved universally vulnerable (49–75% loss), particularly under prolonged hygrothermal exposure where it showed 40% strength reduction after 1500 h. These variations were mechanistically explained through infrared spectroscopy—ester group cleavage at 1700 cm^−1^ correlated with vinyl ester degradation, while molecular chain scission drove polyester failure.

2. Suggestions Related to the Effectiveness of Testing Methods and Test Duration

The DMA method was highly consistent with the results of the traditional static bending test, providing new ideas for evaluating polymer adhesives. The IR spectroscopy method helped us understand the aging mechanism from the perspective of the microscopic molecular structure and assisted with the scientific research and development of adhesives.

All three evaluation methods (bending, DMA, IR) demonstrated convergent results: DMA loss factors in polymer-rich adhesives decreased from >1.0 to stable values after 500 h aging, mirroring bending strength stabilization (±5% variation beyond 500 h). Infrared spectroscopy confirmed that structural stabilization accompanied these macroscopic trends, with no new degradation peaks emerging post-500 h. This confirms that 500 h suffices for accelerated aging assessment, eliminating the need for extended 3000 h tests.

3. Significance of Testing Methods for Research and Future Research Directions

Performance rankings enable targeted adhesive selection: Vinyl ester 4# excels in humid environments (1% hygrothermal loss), while epoxy 3# is optimal for water-exposed structures (15% water-curing loss).

Future research should focus on developing more precise aging prediction models that comprehensively incorporate engineering environmental factors (such as temperature, humidity, and chemical media), adhesive material properties, and time-dependent variables to estimate the service life of adhesives in engineering design accurately. Additionally, efforts should have been directed toward exploring new adhesive formulations that could withstand extreme conditions (e.g., extremely low temperatures in polar regions and high temperatures in deserts) and meet the stringent requirements for ultra-long-life structures (such as century-old buildings and permanent bridges). This would have provided a more reliable material foundation for future engineering projects.

## Figures and Tables

**Table 1:** Instruments used in this study and manufacturers they were sourced from.

Test Instrument	Manufacturer
401 Type Thermal Aging Test Chamber	Shanghai Experimental Instrument General Factory, Shanghai, China
SDH001U Type Damp-Heat Aging Test Chamber	Chongqing Wanda Instrument Co., Ltd., Chongqing, China
Single-row Two-hole Water Bath Test Chamber	Beijing Zhongxing Weiye Instrument Co., Ltd., Beijing, China
AG—IS 100kN Universal Testing Machine	Shimadzu Corporation, Kyoto, Japan
DMA 242C Type Dynamic Mechanical Analyzer	NETZSCH Group, Selb, Germany
TENSOR 27 Type Fourier Transform Attenuated Total Reflection Infrared Spectrometer	BRUKER OPTIK GMBH, Ettlingen, Germany

**Table 2:** Aging rates of adhesive samples under different aging conditions.

Sample Number	Alkali Treatment	Heat–Moisture Treatment	Water-Curing Treatment
Aging time	3000 h	3000 h	480 h
1#	28	20	59
2#	40	16	30
3#	27	0	15
4#	19	1	22
5#	70	51	77
6#	58	49	75

## Data Availability

The original contributions presented in this study are included in the article. Further inquiries can be directed to the corresponding author.
